# Prediction of Arctic plant phenological sensitivity to climate change from historical records

**DOI:** 10.1002/ece3.2702

**Published:** 2017-02-01

**Authors:** Zoe A. Panchen, Root Gorelick

**Affiliations:** ^1^ Department of BiologyCarleton UniversityOttawaONCanada

**Keywords:** Arctic, climate change, flowering time, herbarium specimen, Nunavut, seed dispersal time, temperature sensitivity

## Abstract

The pace of climate change in the Arctic is dramatic, with temperatures rising at a rate double the global average. The timing of flowering and fruiting (phenology) is often temperature dependent and tends to advance as the climate warms. Herbarium specimens, photographs, and field observations can provide historical phenology records and have been used, on a localised scale, to predict species’ phenological sensitivity to climate change. Conducting similar localised studies in the Canadian Arctic, however, poses a challenge where the collection of herbarium specimens, photographs, and field observations have been temporally and spatially sporadic. We used flowering and seed dispersal times of 23 Arctic species from herbarium specimens, photographs, and field observations collected from across the 2.1 million km^2^ area of Nunavut, Canada, to determine (1) which monthly temperatures influence flowering and seed dispersal times; (2) species’ phenological sensitivity to temperature; and (3) whether flowering or seed dispersal times have advanced over the past 120 years. We tested this at different spatial scales and compared the sensitivity in different regions of Nunavut. Broadly speaking, this research serves as a proof of concept to assess whether phenology–climate change studies using historic data can be conducted at large spatial scales. Flowering times and seed dispersal time were most strongly correlated with June and July temperatures, respectively. Seed dispersal times have advanced at double the rate of flowering times over the past 120 years, reflecting greater late‐summer temperature rises in Nunavut. There is great diversity in the flowering time sensitivity to temperature of Arctic plant species, suggesting climate change implications for Arctic ecological communities, including altered community composition, competition, and pollinator interactions. Intraspecific temperature sensitivity and warming trends varied markedly across Nunavut and could result in greater changes in some parts of Nunavut than in others.

## 1 Introduction

The timing of flowering and fruiting (phenology) is often influenced by temperatures in the month or two preceding flowering or fruiting (Fitter, Fitter, Harris, & Williamson, 1995; Panchen & Gorelick, 2015; Panchen, Primack, Aniśko, & Lyons, 2012). Phenological temperature sensitivity has been used to identify plants that are indicators of climate change and the responsiveness of plants to climate change (Bertin, 2015; Gallagher, Leishman, & Hughes, 2009; Menzel et al., 2006; Panchen et al., 2012; Rumpff, Coates, & Morgan, 2010; Springate & Kover, 2014). Herbarium specimens, pressed plants often collected in flower or fruit, provide a reliable historical record of flowering and fruiting phenology for use in phenology–climate change studies (Davis, Willis, Connolly, Kelly, & Ellison, 2015). Many herbarium specimen studies from temperate regions have been used to study flowering time responses to contemporary climate change (Davis et al., 2015; Diskin, Proctor, Jebb, Sparks, & Donnelly, 2012; Gallagher et al., 2009; Hart, Salick, Ranjitkar, & Xu, 2014; Lavoie & Lachance, 2006; MacGillivray, Hudson, & Lowe, 2010; Munson & Sher, 2015; Neil, Landrum, & Wu, 2010; Panchen et al., 2012; Park & Schwartz, 2015; Primack, Imbres, Primack, Miller‐Rushing, & Del Tredici, 2004; Robbirt, Davy, Hutchings, & Roberts, 2010). There are, however, few studies on the effects of climate change on the timing of fruiting events (Gallinat, Primack, & Wagner, 2015) and, to our knowledge, no studies that have used herbarium specimens to assess the impacts of climate change on timing of seed dispersal nor on flowering and seed dispersal times of Arctic plants. It is important to study multiple life history stages because phenological responsiveness to climate change can vary across life history stages (Post, Pedersen, Wilmers, & Forchhammer, 2008). The Arctic is experiencing unprecedented climate change with temperatures rising at a rate double the global average (AMAP, 2012a; Furgal & Prowse, 2007; McBean, 2004; Przybylak, 2003) and hence the importance of understanding Arctic plant phenological responses to climate change.

In temperate regions, herbarium specimens have often been collected regularly on a local scale enabling the construction of a flowering phenology time series at a single location over extended periods of time, and hence, most temperate phenology–climate change studies have focused on a localised area with homogeneous topography and climatology. In situations where there are spatial or temporal gaps in the phenology record from herbarium specimens, the phenological historical records have been successfully augmented with dated photographs and field observations (Bertin, 2015; MacGillivray et al., 2010; Miller‐Rushing, Primack, Primack, & Mukunda, 2006; Panchen et al., 2012; Robbirt et al., 2010). Conducting a similar study in the Arctic, however, poses a challenge (Holopainen, Helama, Lappalainen, & Gregow, 2013). Herbarium specimens, photographs, and field observations have only been collected sporadically and, on many occasions, only once from a particular location across the whole of the topographically and climatologically varied Nunavut territory, Canada, necessitating a study on large spatial scales. The largest area, to date, used in herbarium specimen climate change phenology analysis is in Ohio, where a 116,000 km^2^ area with 26 weather stations was assessed (Calinger, Queenborough, & Curtis, 2013). Nunavut has an area of 2.1 million km^2^ and just 11 weather stations with long‐term temperature records. In addition, almost all of the weather stations in Nunavut are coastal and hence influenced by the effect of the sea ice and its melting regime and therefore may not be reflective of temperatures in the interior (Atkinson & Gajewski, 2002).

Long‐term studies of the temperature sensitivity of Arctic plant flowering and fruiting times are limited (Cadieux et al., 2008; Ellebjerg, Tamstorf, Illeris, Michelsen, & Hansen, 2008; Iler, Hoye, Inouye, & Schmidt, 2013a; Panchen & Gorelick, 2015; Thórhallsdóttir, 1998). However, there have been a number of experimental warming studies on Arctic flowering phenological sensitivity to warming temperatures, indicating that many Arctic plants advance flowering in warmer temperatures (Alatalo & Totland, 1997; Bjorkman, Elmendorf, Beamish, Vellend, & Henry, 2015; Jones, Bay, & Nordenhall, 1997; Khorsand Rosa et al., 2015; Oberbauer et al., 2013; Stenström, Gugerli, & Henry, 1997; Welker, Molau, Parsons, Robinson, & Wookey, 1997), but there is evidence that such studies underestimate the phenological impact of a warming climate (Wolkovich et al., 2012). The observed climate change in the Arctic is predominantly in late summer, autumn, and winter which may favour advancing seed dispersal phenology over advancing flowering phenology (AMAP, 2012a; Furgal & Prowse, 2007; McBean, 2004; Panchen & Gorelick, 2015). Other factors that can be correlated with the time of flowering are photoperiod and snow melt‐out date (Bernier & Périlleux, 2005; Inouye, Saavedra, & Lee‐Yang, 2003; Rathcke & Lacey, 1985), but temperature appears to be the key driver in the timing of flowering of Arctic and alpine plants (Hülber, Winkler, & Grabherr, 2010; Keller & Körner, 2003; Thórhallsdóttir, 1998).

The primary objectives of this research were to use herbarium specimens, photographs, and field observations collected from across Nunavut to determine (1) which monthly temperatures most strongly influence the timing of flowering and timing of seed dispersal of Arctic plants; (2) the sensitivity of Arctic plant flowering times and seed dispersal times to temperature as an indicator of the impact of climate change on Arctic plant phenology; and (3) whether there has been a change in flowering times and seed dispersal times over the last 120 years in Nunavut. A complementary objective was to assess contemporary climate change with regard to changes in monthly temperatures in Nunavut. More broadly, this research will serve as a proof of concept to assess whether phenology–climate change studies using historic data can be conducted at large spatial scales.

## 2 Materials and Methods

### 2.1 Flowering time and seed dispersal time data

**Figure 1:**
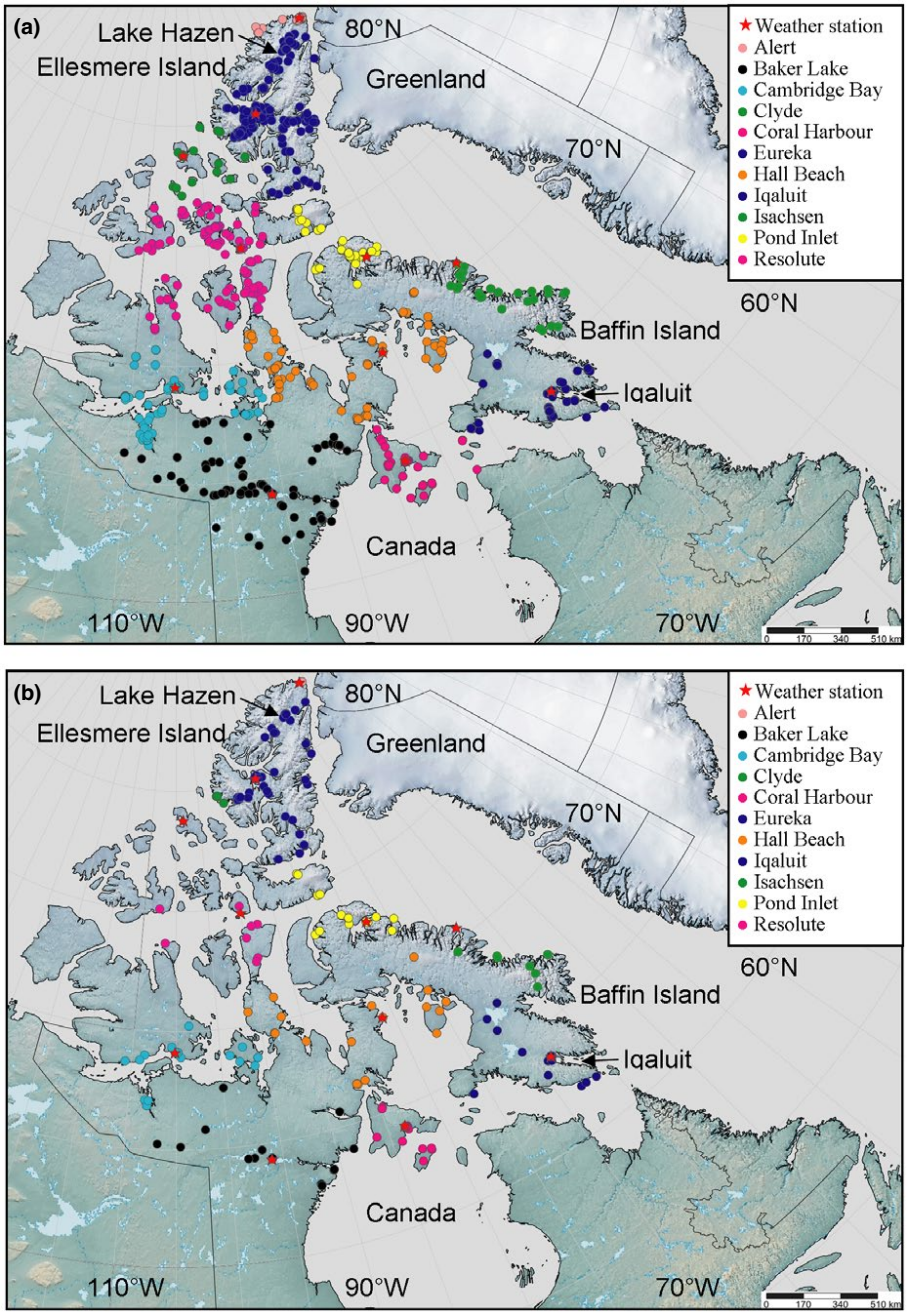
Locations of (a) flowering and (b) seed dispersing collections (1946–2015) color coded by the assigned weather station for each location

**Figure 2:**
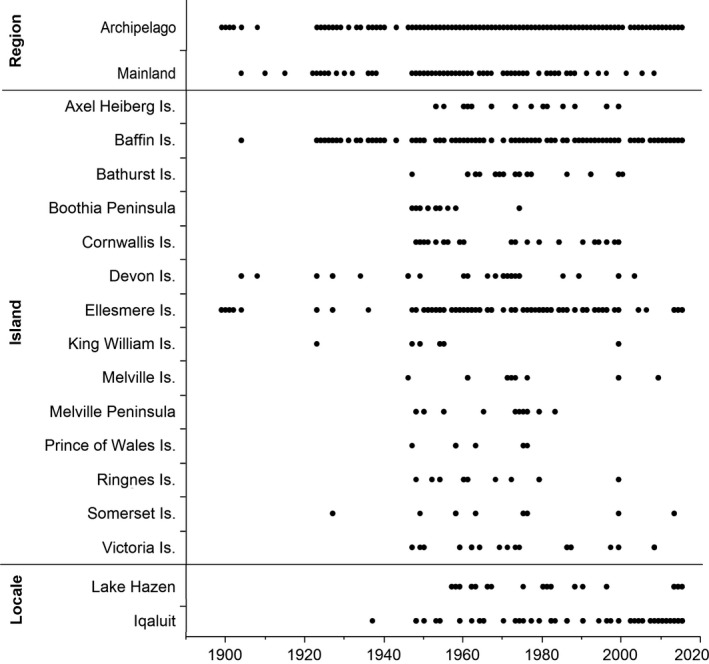
Years in which collections were made of flowering and dispersing seed herbarium specimens, photographs, and field observations from the Nunavut mainland and Nunavut archipelago regions, Nunavut archipelago islands and peninsulas, and the Lake Hazen and Iqaluit locales. The black markers indicate years in which one or more collections were made

To determine the flowering and seed dispersal times of 23 common Nunavut Arctic plant species (Table 1) over the past 120 years, we examined herbarium specimens collected from across Nunavut, Canada, from 1896 to 2015 (Table S1). We also included in the dataset flowering and seed dispersal times from field observations at both Lake Hazen, Quttinirpaaq National Park, Ellesmere Island, and Iqaluit, Baffin Island, Nunavut, in 2013–2015 (Panchen, 2016; Panchen & Gorelick, 2016) and photographs from the Canadian Museum of Nature's photographic collection and private photographic collections (Table S1). We excluded from the dataset herbarium specimens and photographs that were any of the following: south of the tree line, west of longitude 111°W, duplicate herbarium specimens or photographs, or any records of plants not in flower or not dispersing seed. For each herbarium specimen, field observation, or photograph (henceforth referred to as a collection data point), we recorded the phenological state (flowering or dispersing seed), collection date representing the time of flowering or time of seed dispersal in number of days from 1st January (henceforth referred to as flowering day of year [DOY] or dispersing seed DOY), year of collection, and latitude and longitude of the collection data point location. The sample size for all collection data points was 3,795, with 3,353 in flower and 442 dispersing seed. For the field observations, the population's mean peak flowering or peak seed dispersal date at a site was used as the collection date. The “flowering” phenology state was when the petals were open, i.e., not in a bud, the petals looked fresh and were not wilted or discoloured, and the stigmas and anthers were visible. The “dispersing seed” phenology state was when the fruit had dehisced or the styles were extended and untwisted (*Dryas integrifolia* L.) or the capitulum had formed into a spherical seed head (*Asteraceae* species). There were no dispersing seed collection data points for *Diapensia lapponica* L., *Saxifraga cernua* L., and *Tofieldia pusilla* (Michx.) Pers. In order to compare the phenological sensitivity to temperature in different parts of Nunavut and at different spatial scales, we classified each collection data point by region (Nunavut mainland or Nunavut archipelago), by island (for Nunavut archipelago collection data points only), and by locale (for Lake Hazen or Iqaluit collection data points only; Figures 1 and 2). Islands north of Hudson Bay, and Boothia and Melville Peninsulas were classified as Nunavut archipelago. Islands further south and in Hudson Bay were classified with the latitudinally and climatically comparable Nunavut mainland (Canadian Ice Service, 2002).

**Table 1:** Mean, standard deviation, minimum, maximum, and range of flowering day of year (DOY) over the past 120 years (1896–2015) of 23 plant species as determined from herbarium specimens, photographs, and field observations collected from across Nunavut, Canada

Species	Mean flower DOY	*N*	Std Dev	Min DOY	Max DOY	Range
*Erysimum pallasii* (Pursh) Fern.	182.6	58	9.1	163	206	43
*Saxifraga oppositifolia* L.	186.3	282	15.8	145	229	84
*Androsace septentrionalis* L.	187.3	34	11.4	164	211	47
*Erigeron compositus* Pursh	192.2	48	12.9	172	227	55
*Ranunculus nivalis* L.	192.6	115	19.0	155	243	88
*Eutrema edwardsii* R. Br.	194.8	123	12.6	157	227	70
*Diapensia lapponica* L.	195.7	57	12.8	173	228	55
*Pedicularis hirsuta* L.	195.8	207	12.1	171	233	62
*Pedicularis flammea* L.	196.1	71	10.4	177	225	48
*Dryas integrifolia* Vahl	196.4	280	13.8	168	233	65
*Ranunculus sulphureus* Sol.	197.2	155	13.8	166	237	71
*Pedicularis arctica* R. Br.	197.7	109	12.9	171	226	55
*Pedicularis capitata* Adams	199.9	126	11.0	175	226	51
*Tofieldia pusilla* (Michx.) Pers.	202.1	60	8.0	183	220	37
*Pedicularis lapponica* L.	202.3	78	12.3	173	237	64
*Arnica angustifolia* Vahl	202.7	124	13.6	172	237	65
*Saxifraga flagellaris* Willd.	203.8	133	14.5	174	239	65
*Saxifraga tricuspidata* Rottb.	204.2	227	13.3	172	243	71
*Saxifraga cespitosa* L.	204.6	340	14.6	164	246	82
*Chamerion latifolium* (L.) Holub	205.2	195	10.6	180	237	57
*Saxifraga cernua* L.	210.0	260	14.0	172	252	80
*Saxifraga hirculus* L.	210.6	201	15.1	172	245	73
*Saxifraga aizoides* L.	212.7	70	12.6	188	240	52

The process we used to choose the 23 species for this study was as follows. First, species with at least 50 herbarium specimens in flower were selected to ensure a large enough sample size. Second, species whose taxonomy was in doubt were eliminated from the analysis. Wind pollinated species were also eliminated because anthesis or receptive stigma are rarely captured or easy to identify on a herbarium specimen. Third, using our phenology monitoring data from Lake Hazen and Iqaluit, species with long flowering durations (>3 weeks), e.g., *Cassiope tetragona* (L.) D. Don which flowers for 3–4 weeks (Panchen, 2016; Panchen & Gorelick, 2016), were eliminated because there would be large variance in flowering DOY. Species where it was difficult to determine whether the plant was in flower, e.g., *Oxyria digyna* (L.) Hill, were also eliminated from the analysis.

### 2.2 Temperature data

For the 11 Nunavut weather stations with continuous or close to continuous data from 1946 to 2015 (Figure 1), we extracted monthly mean temperatures directly from Environment Canada's national climate data archive (Environment Canada, 2016) or calculated monthly mean temperatures from Environment Canada's daily temperature archive data. In some instances, the monthly temperatures were missing from the Environment Canada data and, in these cases, we hindcast or reconstructed the monthly mean temperature using data from the closest weather station (Leathers, Malin, Kluver, Henderson, & Bogart, 2008; Panchen & Gorelick, 2015; Panchen et al., 2012; Throop, Smith, & Lewkowicz, 2010). The latitude, longitude, and elevation of the weather stations have not changed over the 70‐year period. Each collection data point was associated with the nearest, most climatically logical weather station based on synoptic and sea ice regimes (Canadian Ice Service, 2002; Fletcher & Young, 1970; Fraser, 1983) and hence with that weather stations’ monthly mean temperatures in the year of collection (Figure 1).

### 2.3 Analysis

To determine which monthly temperatures are most strongly correlated with the time of flowering of Arctic plants across Nunavut, we ran a standard least squares mixed model with flowering DOY as the response variable, species as a random effect and May, June, July, and August mean temperatures as fixed effects. We repeated this model run separately for each region, each island (Baffin and Ellesmere Islands only), and each locale (Lake Hazen and Iqaluit only), using Nunavut mainland, Nunavut archipelago, Baffin Island, Ellesmere Island, Lake Hazen, or Iqaluit flowering collection data points. We ran a similar set of models to determine which monthly temperatures are most strongly correlated with the time of seed dispersal with dispersing seed DOY as the response variable. Baffin Island and Ellesmere Island were chosen from the island classification because they were the only islands with regular collections since 1920 for Baffin Island and since 1957 for Ellesmere Island (Figure 2).

To determine sensitivity of Arctic plant flowering times to temperature, we ran linear regressions for each species from across Nunavut separately with flowering DOY as the response variable and June mean temperature as the explanatory variable. We repeated the regression analyses separately for each region, island, and locale in order to compare the flowering time temperature sensitivity of plants on the Nunavut mainland versus conspecific plants on Nunavut archipelago and similarly Baffin Island plants versus Ellesmere Island conspecifics, and Lake Hazen plants versus Iqaluit conspecifics. There were insufficient data to determine sensitivity of Arctic plant seed dispersal times to temperature per species; hence, we used a standard least squares mixed model to determine seed dispersal time temperature sensitivity across Nunavut to July mean temperature across the 20 species with dispersing seed DOY as the response variable, July mean temperature as the fixed effect, and species as a random effect, and repeated for Nunavut archipelago, Baffin Island, and Ellesmere Island where there were sufficient data.

To determine whether there has been a trend toward earlier flowering times over the past 120 years (1896–2015) across Nunavut, we ran a standard least squares random intercept mixed model with flowering DOY as the response variable, species as a random effect, and year as a fixed effect. We ran a similar model to determine whether there has been a trend toward earlier seed dispersal times over the past 120 years (1896–2015), with dispersing seed DOY as the response variable.

To test whether there was a bias in collection dates toward earlier herbarium specimen collection in more recent years, we correlated the date of all herbarium specimens collected for all 23 species against the year of collection (1896–2015) and for each species individually for the years 1946–2015. We used these year ranges combined with across species (1896–2015) and individual species (1946–2015) to match the analyses of change in flowering/seed dispersal time over time (1896–2015) and change in flowering with temperature per species (1946–2015). We used all herbarium specimens in the correlations, including those that were not in flower or dispersing fruit, to reflect when collections were made over the years. We ran these correlations using the National Herbarium of Canada (CAN) data because this collection has the most extensive and comprehensive collection of Nunavut herbarium specimens and the collection is completely databased (Table S1).

To assess temperature changes in Nunavut, we correlated monthly mean and annual mean temperatures versus year (1946–2015) for the 11 weather stations. Since there might have been a regime shift over this time period with a cooling period followed by a warming period (AMAP, 2012b; McBean, 2004; Przybylak, 2003; Reid et al., 2015; Throop et al., 2010), we also conducted change point analyses for each of the 11 weather stations for each of annual, June, and July mean temperatures separately using a nonlinear least squares model with a prediction formula for the change point of (*B*0 + (*B*1 × Year) + (*B*2 × (If Year ≥ *C*, Then (Year − *C*) else 0))). All statistical analysis was conducted using JMP12 (SAS Institute, Cary, NC, USA).

## 3 Results

**Figure 3:**
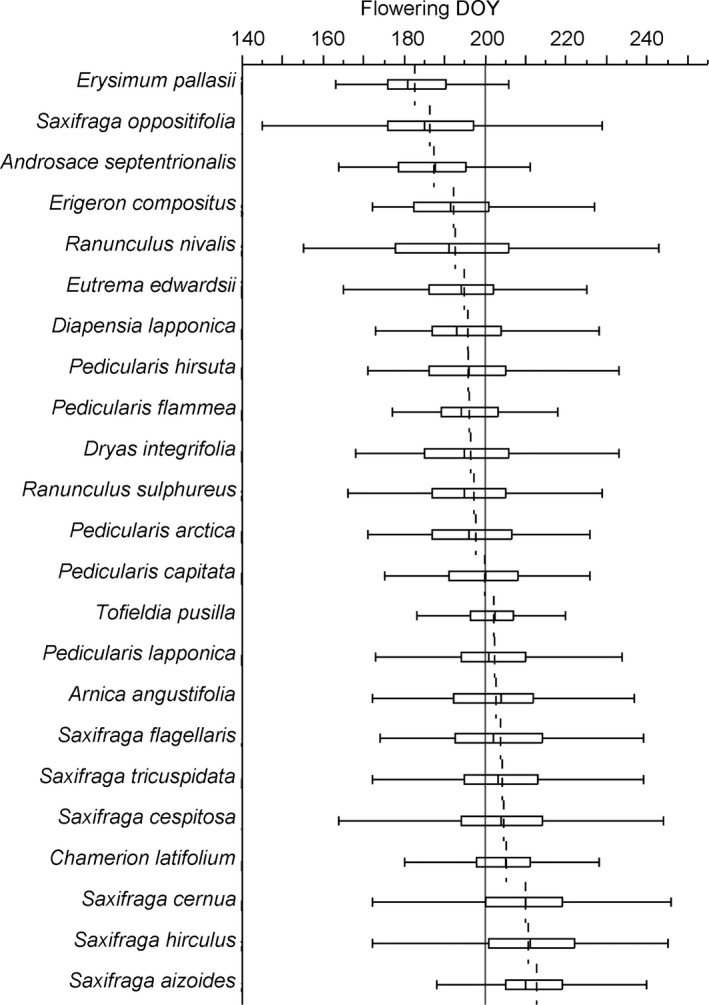
Range of flowering day of year (DOY) of the 23 species in this study as recorded on the herbarium specimens, photographs and field observations.. Each box plot shows the species’ flowering DOY quartiles, the dotted line is the species’ mean flowering DOY, and the solid line is the mean flowering DOY across species

There is considerable variation in the range of flowering DOY of each species over the 120 years (Table 1, Figure 3). The species with the least variation was *Erysimum pallasii* (Pursh) Fernald, with a range of 43 days. The species with the greatest variation in flowering DOY was the snow bed species *Ranunculus nivalis* L., with a range of 88 days. The order of flowering (Figure 3) is consistent with recent observations (Panchen & Gorelick, 2016), indicating that the collection flowering time data are representative of species’ relative time of flowering through the growing season.

June mean temperature had the strongest correlation with the timing of flowering at all spatial scales, except Ellesmere Island where July mean temperature had the strongest correlation (Table 2). May to August mean temperatures also had a significant correlation with the timing of flowering at some spatial scales. July mean temperature had the strongest correlation with the timing of seed dispersal at all spatial scales, except Nunavut mainland where, although not significant, August had the strongest correlation (Table 3). As expected, in general the models had better fit at finer‐grained spatial scales.

**Table 2:** Standard least squares mixed model results at different spatial scales with flowering DOY as the response variable, species as a random effect, and May, June, July, and August mean temperatures as fixed effects, showing June mean temperature generally had the strongest correlation with the time of flowering and models have better fit at finer spatial scales

	Overall model			May (°C)		June (°C)		July (°C)		August (°C)	
	Adj *R* ^2^	*N*	RMSE	*F*	*p*	*F*	*p*	*F*	*p*	*F*	*p*
Nunavut	.30	3,022	12.45	6.97	.0083	154.47	<.0001	22.56	<.0001	57.75	<.0001
Nunavut mainland	.23	529	11.74	0.09	.7642	6.88	.0090	0.70	.4027	0.11	.7443
Nunavut archipelago	.32	2,493	12.48	4.23	.0399	133.96	<.0001	32.21	<.0001	36.22	<.0001
Baffin Island	.38	781	12.28	6.82	.0092	62.93	<.0001	2.15	.1428	10.68	.0011
Ellesmere Island	.29	799	10.91	0.68	.4090	4.90	.0272	59.77	<.0001	2.99	.0840
Iqaluit	.61	351	9.42	0.08	.7776	40.37	<.0001	7.00	.0085	6.04	.0145
Lake Hazen	.39	308	8.56	3.15	.0772	10.44	.0014	1.28	.2583	1.42	.2351

**Table 3:** Standard least squares mixed model results at different spatial scales with dispersing seed DOY as the response variable, species as a random effect, and May, June, July, and August mean temperatures as fixed effects, showing July mean temperature generally had the strongest correlation with time of seed dispersal and models have better fit at finer spatial scales

	Overall model			May (°C)		June (°C)		July (°C)		August (°C)	
	Adj *R* ^2^	*N*	RMSE	*F*	*p*	*F*	*p*	*F*	*p*	*F*	*p*
Nunavut	.23	346	11.42	0.04	.8391	0.61	.4342	41.33	<.0001	21.96	<.0001
Nunavut mainland	.45	58	10.01	0.25	.6171	0.06	.8063	0.04	.8428	2.50	.1200
Nunavut archipelago	.26	288	11.29	0.51	.4760	0.14	.7099	48.20	<.0001	29.30	<.0001
Baffin Island	.24	123	11.29	0.19	.6652	1.32	.2537	14.69	.0002	0.14	.7071
Ellesmere Island	.19	87	9.62	6.39	.0134	0.75	.3884	15.49	.0002	9.14	.0034
Iqaluit	.63	65	8.99	27.35	<.0001	5.30	.0254	46.96	<.0001	0.09	.7648
Lake Hazen	.29	47	5.38	0.06	—	1.20	—	0.69	—	0.03	—

**Figure 4:**
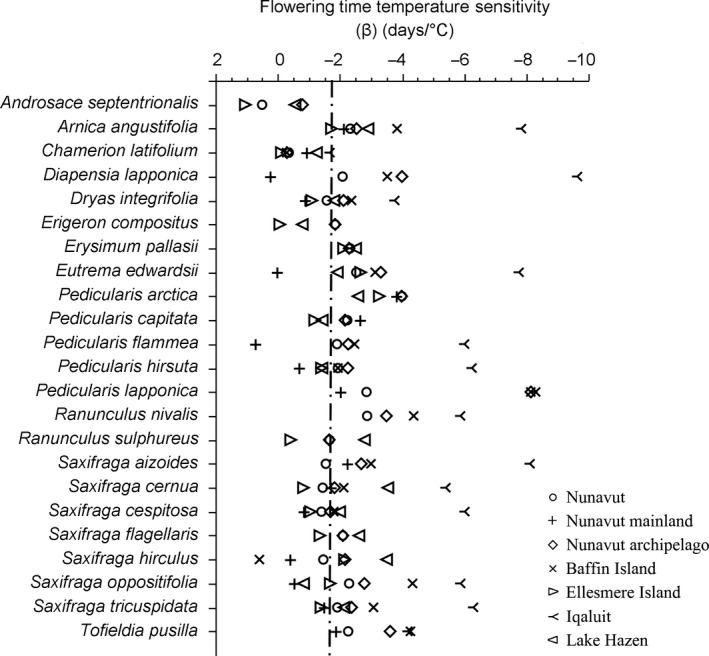
Species’ flowering time temperature sensitivity (β) at different spatial scales in Nunavut, Canada. Significant sensitivity is to the right of the dashed vertical line (Table S2)

All but two of the 23 species showed a significant negative relationship between time of flowering and June mean temperature, that is, these species flower earlier with warmer June mean temperatures (Figure 4, Table S2). The magnitude of a species’ flowering time sensitivity to June mean temperature varied across Nunavut. The flowering phenology of plants in the Nunavut archipelago was generally more sensitive to June mean temperatures than conspecific plants on the Nunavut mainland, and plants on Baffin Island were generally more sensitive than conspecifics on Ellesmere Island. Flowering times at Iqaluit were generally the most sensitive to June mean temperature. Flowering time temperature sensitivity varied dramatically ranging from −1.7 days/°C (*S. cernua* L. on Nunavut mainland) to −9.6 days/°C (*D. lapponica* at Iqaluit). The annual/biennial *Androsace septentrionalis* and the late‐flowering *Chamerion latifolium* (L.) Holub were the only species whose flowering time showed no sensitivity to temperature. The seed dispersal time sensitivity to July mean temperature of the 20 species from across Nunavut was −1.79 days/°C (*N* = 346, *p* < .0001). That is, seed dispersal was 1.79 days earlier for every 1°C rise in July mean temperature. The seed dispersal time sensitivity to July mean temperature across species was −2.3, −3.65, and −1.64 days/°C in Nunavut archipelago, Baffin Island, and Ellesmere Island, respectively (*N* = 288, 123, 87, respectively, *p* < .0001).

**Figure 5:**
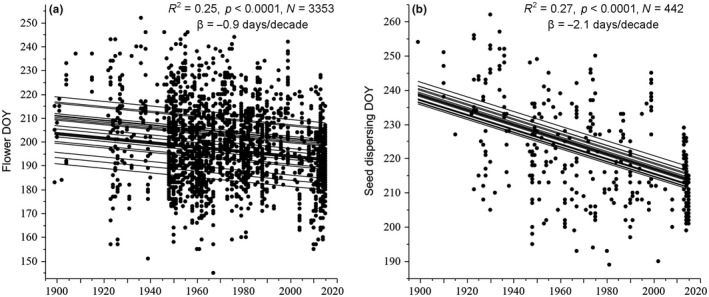
Standard least squares random intercept mixed model with (a) flowering DOY (day of year) and (b) dispersing seed DOY as the response variable, species as a random effect, and year as a fixed effect across 23 species (a) and 20 species (b) in Nunavut where β is the days/decade change in flowering or seed dispersal time and trend lines represents the best fit for each species

Across Nunavut, plants flowered 0.9 days/decade earlier over the past 120 years (1896–2015; *R* ^2^ = .25, *N *=* *3,353, *p *<* *.0001; Figure 5a) but dispersed seed 2.1 days/decade earlier over the 120 years (*R* ^2^ = .27, *N *=* *442, *p *<* *.0001; Figure 5b).

The correlation of collection date for all herbarium specimens versus year (1896–2015) was very weak (*R* ^2^ = .05 *N* = 3,025, *p* < .0001). There was no significant correlation per species between collection date for all herbarium specimens and year (1946–2015) for most species (Table S3). This suggests there was little to no change in collection time frame over the years and unlikely to have caused a bias in our analysis.

Annual temperatures have risen significantly since 1946 at nine of the 11 weather stations, albeit with a very weak correlation at Hall Beach (Nunavut archipelago) and Pond Inlet (Baffin Island), while annual temperatures at Clyde (Baffin Island) and Iqaluit (Baffin Island) have not risen significantly (Table 4). Baker Lake (Nunavut mainland) and Cambridge Bay (Victoria Island) in the south and west of Nunavut experienced the most dramatic annual temperature increases of 0.30 and 0.35°C/decade, respectively. In contrast, May and August mean temperatures have not risen significantly at any of the 11 weather stations. June mean temperatures have risen significantly since 1946 only at Baker Lake, Cambridge Bay, and Pond Inlet and, although significant, very weakly correlated at Coral Harbour (Nunavut mainland; 0.36, 0.33, 0.25, and 0.24°C/decade, respectively). Following a similar pattern to the June mean temperature, July mean temperature has risen significantly since 1946 at Baker Lake, Cambridge Bay, Coral Harbour, Eureka (Ellesmere Island), Pond Inlet, and, although significant, are very weakly correlated at Clyde (0.28, 0.30, 0.37, 0.26, 0.37, and 0.17°C/decade, respectively). The most dramatic June mean temperature increases are at Baker Lake and Cambridge Bay with 0.36 and 0.33°C/decade rise, respectively, while the most dramatic July mean temperature increases are at Coral Harbour and Pond Inlet, both rising 0.37°C/decade.

**Table 4:**
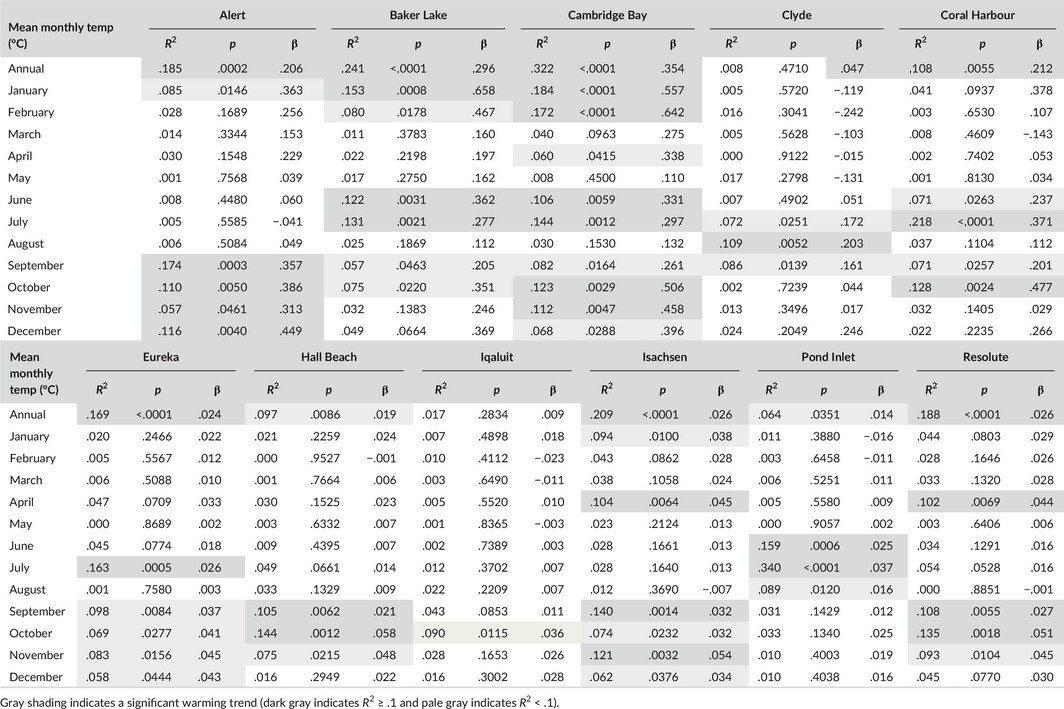
Correlation of monthly mean or annual mean temperatures versus year (1946–2015) for the 11 long‐term weather stations in Nunavut, Canada, where β is the rate of change in monthly temperature in °C/yr

**Figure 6:**
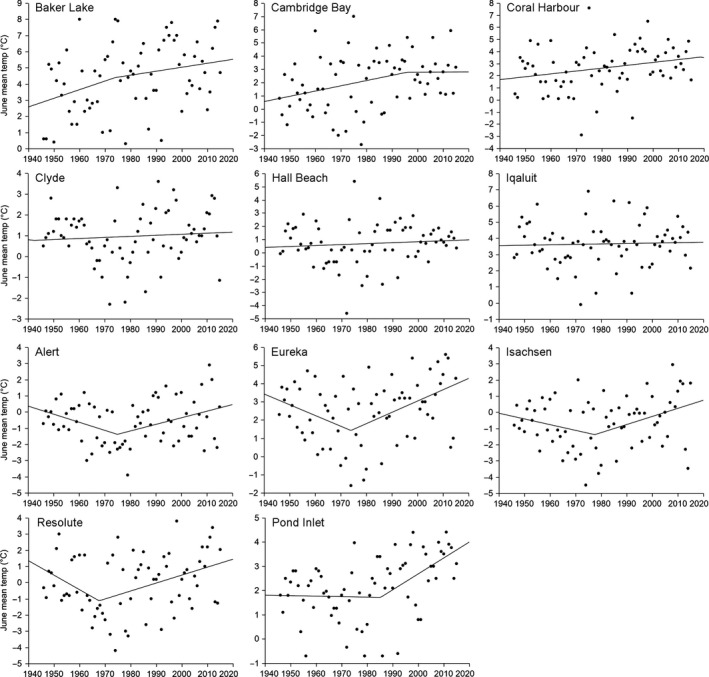
June mean temperatures since 1946 with regime shift trend line for the 11 long‐term weather stations in Nunavut, Canada (Table S2). Baker Lake, Cambridge Bay, and Coral Harbour have experienced continually rising temperatures in June since 1946; Clyde, Hall Beach, and Iqaluit have experienced no significant warming in June since 1946; Alert, Eureka, Isachsen, and Resolute have experienced a regime shift from a cooling period to a warming period in June and Pond Inlet has experienced a regime shift from a steady temperature to a warming period in June

Since 1946, a regime shift has been experienced at Alert, Eureka, Isachsen, and Resolute (Nunavut archipelago) weather stations, with a cooling or steady temperature period followed by a warming period with change points in the 1970s to 1980s for annual mean temperatures, the late 1960s and early 1970s for June mean temperatures, and 1990s to 2000s for July mean temperatures (Figure 6, Table S4). Baker Lake and Coral Harbour (Nunavut mainland) and Cambridge Bay (Victoria Island) weather stations experienced an annual mean temperature regime shift from steady temperatures to warming temperatures in 1987, 1964, and 1989, respectively, but no significant regime shift for June or July mean monthly temperatures. Clyde, Hall Beach, Iqaluit, and Pond Inlet weather stations experienced an annual mean temperature regime shift from cooling or steady temperatures to warming temperatures, but only Pond Inlet has seen a June and July mean temperature regime shift from cooling to warming temperatures in 1985 and 1977, respectively. Large interannual variation in monthly and annual temperatures of several degrees Celsius was observed for all weather stations (Figure 6).

## 4 Discussion

Flowering time sensitivity to June temperatures varied dramatically among the 23 Nunavut Arctic plant species and intraspecifically across different parts of Nunavut. Intraspecifically, flowering time sensitivity was greater in the Arctic Archipelago than on the Nunavut mainland and similarly Baffin Island plants were more sensitive than their conspecifics on Ellesmere Island. The intraspecific variation in temperature sensitivity could be indicative of adaptation to different climatic conditions across Nunavut and smaller temperature changes in colder location having a relatively larger temperature sensitivity due to lower growing degree days required to flower at colder locations (Panchen & Gorelick, 2016; Parmesan, 2007; Prevéy et al., In Press). The diverse intraspecific flowering time sensitivity to temperature in different parts of Nunavut and the variation in warming trends in different parts of Nunavut (Tables 4, S2 and Figure 6) suggests that there could be greater changes in some parts of Nunavut than in others. From the warming trend and temperature sensitivity analysis conducted here, the greatest and most immediate changes are likely to be seen in the south and west, i.e., Nunavut mainland and Victoria Island, with Victoria Island likely to see the greatest changes because of the greater temperature sensitivity on the Nunavut archipelago than the Nunavut mainland. The least changes could be seen on Baffin Island; however, this could be counterbalanced by the apparent greater flowering time temperature sensitivity of Baffin Island plants. As the Arctic climate warms, the variation in flowering and fruiting time sensitivity to temperature among species and intraspecifically has implications for Arctic ecological communities, including altered community composition, competition, and pollinator interactions (Callaghan, 2005; CaraDonna, Iler, & Inouye, 2014; Ellebjerg et al., 2008; Euskirchen, Carman, & McGuire, 2014; Hegland, Nielsen, Lázaro, Bjerknes, & Totland, 2009; Høye, Post, Schmidt, Trøjelsgaard, & Forchhammer, 2013; McKinney et al., 2012; Molau, Nordenhäll, & Eriksen, 2005; Parmesan, 2007; Rathcke & Lacey, 1985).

Given that (1) flowering times and fruiting times are most correlated with June and July temperatures, respectively and (2) compared to June temperatures, July temperatures are warming more and warming across a wider area of Nunavut, it is not surprising that seed dispersal times have advanced over twice as fast as flowering times over the past 120 years in Nunavut. This implies that the duration for seeds to mature is becoming shorter and there is potential for greater sexual reproductive success and an extended reproductive season in the short Arctic growing season (Alatalo & Totland, 1997; Klady, Henry, & Lemay, 2011; Molau, 1993, 1997; Müller, Cooper, & Alsos, 2011; Post et al., 2008; Wookey et al., 1993). Temperatures in Nunavut are rising predominantly at the end of the growing season and during winter, and hence, it might be expected that fruiting times may advance more than flowering times (Panchen & Gorelick, 2015).

As expected, the smaller the spatial scale, the better the model fit. However, even at the largest spatial scale, i.e., across the 2.1 million km^2^ of Nunavut, there was a significant relationship between flowering time or seed dispersal time versus monthly mean temperatures. This is surprising given the large geographical area, the large distances between temperature data sources and different year‐to‐year variations in the synoptic weather systems across Nunavut (Fletcher & Young, 1970; Fraser, 1983; Furgal & Prowse, 2007). Given the large geographical area included in the analysis, the absolute values of the phenological temperature sensitivity should be treated with caution; it is the relative values that are important here. Among the spatial scale comparisons, the flowering time temperature sensitivity of plants at Iqaluit appears to be the most pronounced, but this analysis is on a small spatial scale and hence perhaps temperature sensitivity is underestimated at the larger spatial scales due to greater variations in the flowering times. Similarly, flowering phenology of plants at Lake Hazen appears to be more temperature sensitive than conspecifics from across Ellesmere Island. The start and end year used in temperature climate change analysis, combined with a greater interannual temperature variation than the warming trend, can play a strong role in the magnitude of the warming or phenological trends observed (Baker, Hartley, Butchart, & Willis, 2016; Panchen & Gorelick, 2015).

Different species are known to have different flowering time temperature sensitivity, and, thus, variation among species is to be expected (Calinger et al., 2013; Hart et al., 2014; Kimball, Davis, Weihrauch, Murray, & Rancourt, 2014; Ledneva, Miller‐Rushing, Primack, & Imbres, 2004; Mazer et al., 2013; Miller‐Rushing & Primack, 2008; Panchen et al., 2012; Parmesan, 2007). However, the magnitude of the variation is surprisingly high in contrast to other studies (Oberbauer et al., 2013; Wolkovich et al., 2012) but not unprecedented (Olsson & Ågren, 2002; Wagner & Simons, 2009). Future research could expand on this study to include a larger number of species in order to compare flowering time sensitivity to temperature across life history strategies (Calinger et al., 2013; Molau et al., 2005; Post et al., 2008). Seed dispersal time of the 20 Arctic species also appears to be sensitive to temperature, in contrast to experimental warming studies (Bjorkman et al., 2015; Jones et al., 1997) but in alignment with faster fruit maturation at Zackenberg, Greenland experimental warming sites (Ellebjerg et al., 2008). Only two species, *Androsace septentrionalis* and *Chamerion latifolium*, showed no flowering time sensitivity to June temperatures in any part of Nunavut. *A. septentrionalis* is an annual, or more often biennial in Nunavut, that must complete its life cycle within the year and whose time of flowering is influenced primarily by snow melt date (Inouye et al., 2003). *A. septentrionalis* also showed no significant trend to earlier flowering in an alpine community (CaraDonna et al., 2014). The late‐summer flowering *C. latifolium* also showed no sensitivity to July or August mean temperatures (data not shown), suggesting that its flowering time may be triggered by day‐length. The two species with the greatest variation in time of flowering, *Saxifraga oppositifolia* and *Ranunculus nivalis*, are either early‐flowering and/or snow bed species, groups of species that have been identified by a long‐term phenology study in Sweden to be most labile in terms of flowering time (Molau et al., 2005). Arctic species’ sequence of flowering is consistent from year to year in Nunavut from 1896 to 2015 and is comparable to the current day (Molau et al., 2005; Panchen & Gorelick, 2016; Figure 3a). Hence, herbarium specimens can be used to determine species’ sequence of flowering.

Flowering times were most correlated with June mean temperatures as might be expected given that the majority of species flower in late June and July and the month(s) preceding flowering typically have the strongest correlation with flowering time (Fitter et al., 1995; Panchen & Gorelick, 2015; Panchen et al., 2012). July and August mean temperatures were also correlated with flowering time, albeit less significantly than June mean temperatures, and this is also to be expected given that flowering continues until the end of August (Table 1). Photoperiod and snow melt‐out date are other factors that can be correlated with the time of flowering (Bernier & Périlleux, 2005; Inouye et al., 2003; Rathcke & Lacey, 1985). The Nunavut archipelago receives 24 hr of daylight per day starting at least 1 month before the earliest flowers are observed, while much of the Nunavut mainland experiences darkness during the growing season. Although the flowering time of some Arctic and alpine species is facultatively photoperiodic (Heide, Pedersen, & Dahl, 1990; Hülber et al., 2010; Keller & Körner, 2003), it, therefore, seems unlikely that photoperiod plays a major role in the time of flowering on Baffin, Ellesmere, and other Nunavut archipelago Islands but could play a role on the Nunavut mainland. There is evidence that the snow melt‐out date is correlated with time of flowering of Arctic plants (Bjorkman et al., 2015; Iler, Høye, Inouye, & Schmidt, 2013b; Molau, 1997; Stenström et al., 1997). However, there are exceptions, particularly in polar deserts where there is minimal snow accumulation over winter (Bienau et al., 2015; Ellebjerg et al., 2008; Molau et al., 2005; Panchen & Gorelick, 2015; Thórhallsdóttir, 1998). Much of the Nunavut archipelago is polar desert and receives very little snow accumulation, while the Nunavut mainland receives considerably more snow (Przybylak, 2003). In addition, snow melt‐out date does not appear to differ much between Baffin and Ellesmere Islands (Panchen & Gorelick, 2016). Therefore, photoperiod and/or snow melt‐out date could account for some of the intraspecific differences in flowering time sensitivity to temperature between the Nunavut mainland and Nunavut archipelago but less likely between Baffin and Ellesmere Islands.

Temperature changes observed since 1946 reflect the three synoptic weather systems that dominate Nunavut. Baker Lake, Cambridge Bay, and Coral Harbour are predominantly influenced by continental systems (Fletcher & Young, 1970; Fraser, 1983) (Figure 6) and are experiencing the greatest rises in temperature, both annually and in the months of June and July, and these temperatures have been rising continually since 1946. Alert, Eureka, Isachsen, and Resolute are predominantly influenced by Arctic Ocean basin systems (Edlund & Alt, 1989; Fletcher & Young, 1970; Fraser, 1983) and experienced a regime shift from a cooling period to a warming period (Reid et al., 2015; Throop et al., 2010). Clyde, Iqaluit, and Hall Beach are influenced by Atlantic Ocean systems and have experienced little or no warming annually or in the months of June and July and no regime shift. Pond Inlet can experience any of the three systems in different years or months and perhaps might explain the regime shift from a steady temperature to a warming period. It is possible that the regime shifts could be an artifact of change in temperature measuring equipment, from manual readings in the early days to automated measurement in more recent years. However, if this were the case, we would have expected to see the regime shift in approximately the same year for all months and annually at a weather station and possibly across the weather stations given that Environment Canada would upgrade all of its weather stations at approximately the same time but the regime shift year varied widely across months and stations (Table S4).

In conclusion, flowering times of Nunavut plants are most strongly correlated with June mean temperature and seed dispersal times are most strongly correlated with July mean temperature. On average over the past 120 years, seed dispersal times have advance twice as fast as flowering times in Nunavut and likely reflect greater increases in July than June mean temperatures. The diversity in flowering time temperature sensitivity among species could result in altered community ecology and those changes could vary in different parts of Nunavut given the variation in temperature trends and intraspecific phenological temperature sensitivity across the territory.

## Conflict of Interest

None declared.
